# Gastric Necrosis After Binge Eating in Bulimia: Recovery From Eating Disorder After Total Gastrectomy

**DOI:** 10.3389/fpsyt.2020.00741

**Published:** 2020-07-31

**Authors:** Najate Achamrah, Sébastien Grigioni, Moïse Coëffier, Nadjib Ainseba, Pierre Déchelotte

**Affiliations:** ^1^Nutrition Department, Rouen University Hospital Center, Rouen, France; ^2^Normandie Univ, UNIROUEN, INSERM UMR 1073, Nutrition, Inflammation et dysfonction de l’axe Intestin-Cerveau, IRIB, Rouen, France; ^3^Clinical Investigation Centre CIC 1404, INSERM and Rouen University Hospital, Rouen, France; ^4^Digestive Surgery, Beauvais Hospital Center, Beauvais, France

**Keywords:** binge eating, bulimia, gastric necrosis, gastrectomy, eating disorders

## Abstract

**Background:**

Gastric necrosis following acute gastric dilatation is rare but more common in females with eating disorders, such as anorexia nervosa or bulimia, during which patients often alternate restriction and binge eating behaviors.

**Case Presentation:**

A 37-year old female patient with a history of 15 years of bulimia nervosa was admitted to the emergency department 24 h after binge eating. Abdominal Computed Tomography imaging showed major gastric distension reaching the pelvis and compressing the digestive organs. Total gastrectomy was required because of gastric necrosis. The patient reported significant reduction in bulimic symptoms after gastrectomy.

**Conclusion:**

We discuss here the possible mechanisms underlying this recovery, including changes in gut-derived factors that could mediate eating behavior changes.

## Background

Bulimia nervosa (BN) is an eating disorder characterized by recurrent episodes of binge eating associated with compensatory behavior, such as purging through self-induced vomiting or laxative misuse (1). Binge eating is also described in the anorexia nervosa (AN) binge-eating subtype, and in the binge-eating disorder often associated with severe obesity. Several complications related to acute gastric dilatation (AGD) have been reported including: gastric rupture (2, 3), vascular compression (4), abdominal aortic occlusion (5–8), obstructive acute renal failure (9), sympathetic and parasympathetic neurological compression (2), pyloric stenosis (10), and also gastric ischemia and necrosis (11–14). Gastric necrosis is rare thanks to the rich stomach collateral blood flow, but often associated with high morbidity and mortality (13). Usually, a total gastrectomy is required. We report here the original case of a gastric necrosis following binge eating, in a patient with BN who recovered from her eating disorder after total gastrectomy. We discuss the possible mechanisms underlying this recovery.

## Case Presentation

A 37-year old female patient was admitted to the emergency room 24 h after binge eating. She had no relevant medical history but described a loss of 10 kg in 1997. At that time, she was 62kg and wanted to lose weight. She started vomiting. She also reported a stressful relationship with her partner at this period. BN remained undiagnosed during 15 years.

**Figure 1:**
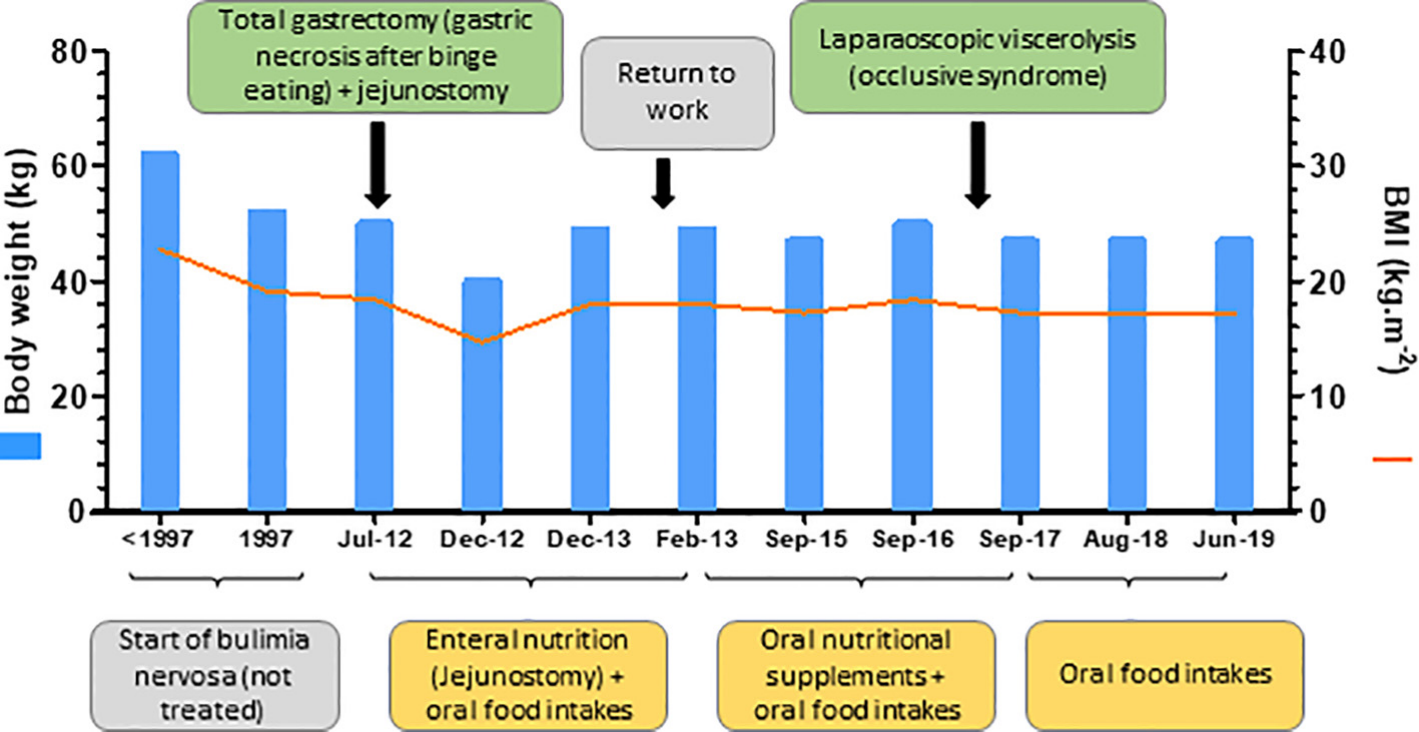
Clinical case timeline.

**Figure 1** shows the timeline of the most relevant events of this clinical case report, and the evolution of weight and body mass index (BMI).

**Figure 2:**
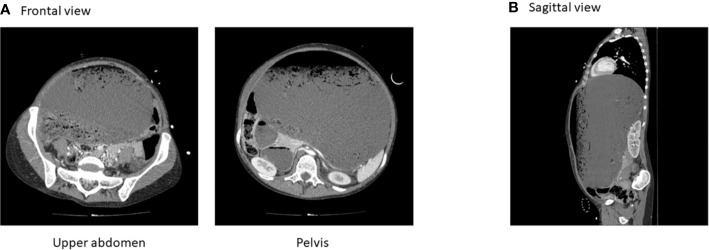
Computed tomography on initial admission. **(A)** Frontal view. **(B)** Sagittal view.

In the emergency room, in July 2012, the patient reported chronic abdominal pain. Physical examination at admission revealed hemodynamic stability, sepsis syndrome, and distended abdomen with defense. Laboratory test results showed high lipasemia and acute renal failure. Abdominal Computed Tomography (CT) imaging showed major gastric distension reaching the pelvis and compressing the digestive organs (**Figure 2**) with no signs of pneumoperitoneum. Conservative gastric decompression was started first using a nasogastric tube aspiration that discharged almost 6.5 litres. Twenty-four hours after hospitalization, the patient exhibited tachycardia and hypotension. Increased doses of Noradrenaline were prescribed unsuccessfully. The patient was prepared for urgent laparotomy showing a large gastric distention associated with necrosis. Total gastrectomy and jejunostomy were performed. The patient was discharged 35 days after the surgical intervention. She was referred to the Department of Clinical Nutrition (Rouen, France) for the nutrition rehabilitation. She weighed 40 kg at this time. Enteral nutrition was administrated through the jejunostomy (1500 kcal/day) and well tolerated. Enteral nutrition was progressively relayed with oral nutritional supplements associated with oral food intakes. The patient reported reduction in bulimic symptoms, structured meals (**Table 1**) and weight stabilisation around 48 kg (body mass index=17.6kg/m²). At the time of first referral in our institution, the patient did not declare any fear for gaining weight or any body shape concern. She was happy with gaining weight thanks to jejunal tube feeding. On following consultations, eating disorders were routinely screened with by the self-administered French version of the SCOFF questionnaire (SCOFF-F) (15). This validated test is routinely used, composed of ﬁve dichotomous questions. One point is given for each “yes” answer. At least two positive answers indicate a positive SCOFF score with a sensitivity of 88.2% and a specificity of 92.5%. This screening consistently resulted in 5 negative answers. One year after total gastrectomy, jejunostomy was finally removed and the patient returned to work. She was admitted to the Digestive surgery department for occlusive syndrome, 5 years after gastrectomy. Laparoscopic viscerolysis has been done, with no complications. The patient has been discharged and maintained sufficient oral food intake. Today, she is still followed-up in the Nutrition unit at least once a year. She still has a negative SCOFF-F score, with abstinence of binge eating and compensatory behaviors (self-induced vomiting, laxative use, diuretics, compensatory exercise, fasting), structured meals and biological markers in normal range (**Table 2**).

**Table 1:** Patient’s food diary.

	Day 1	Day 2	Day 3
Breakfast	Tea, orange, ½ slice of bread	Tea, apple, ½ slice of bread	Tea, orange, ½ slice of bread
Snacks	–	–	–
Lunch	Vegetables, chicken, rice	Vegetables, eggs	Vegetables, potatoes, eggs
Snacks	One oral nutritional supplement	One oral nutritional supplement	One oral nutritional supplement
Dinner	Salad, ½ slice of bread, fish	Vegetables, pasta	Salad, fish, rice
Snacks	–	–	–

**Table 2:** Patient’s plasmatic biological markers.

	Value	Normal ranges
Haemoglobin	12.8 g/l	12.5–15.5 g/l
Hematocrit	0.39	0.34–0.47
Phosphorus	1.45 mmol/l	0.87–1.50 mmol/l
Potassium	4.2 mmol/l	3.5–5 mmol/l
Sodium	140 mmol/l	135–145 mmol/l
Magnesium	0.81 mmol/l	0.75–1 mmol/l
C-reactive protein	<5	<5
Transthyretin	0.28 g/l	0.25–0.35 g/l
Albumin	45.1 g/l	35–50 g/l

## Discussion

Gastric necrosis following acute gastric dilatation (AGD) is rare. AGD due to overeating is more common in females with eating disorders, such as anorexia nervosa or bulimia, during which patients often alternate restriction and binge eating behaviors. Moreover, starvation may induce atony of the stomach which is overtaxed by the rapid eating of large quantity of food, leading to AGD (11) associated with high mortality rate (16). Interestingly, we observed an improvement in the patient’s eating behavior after total gastrectomy, a negative SCOFF-F score and biological parameters in normal range. The BMI (17,6kg/m²) was below the normal range (18.5-25kg/m²), and the patient was still undernourished at that time. But, she did not display any eating disorders behavioral symptoms anymore. Remaining with a lower BMI is common finding after total gastrectomy because of the lack of ghrelin signalling and of altered digestion steps. Her eating disorder has been undiagnosed during 15 years (from 1997 to 2012). Approximately 10% of patients diagnosed with BN will develop chronic illness. There is currently no consensus on the definition of recovery of eating disorders. De Young et al. recently recommended that 6 months be used for recovery definitions of bulimia (17), whereas DSM-V classification provides unspecific guidance, i.e., « criteria not met for a sustained period of time » (1). Richmond et al. reported four items for eating disorders recovery definition according to patients, parents and clinicians: (a) psychological well-being, (b) eating-related behaviors/attitudes, (c) physical markers, and (d) self-acceptance of body image (18).

Mechanisms involved in this recovery are not totally understood but may be close to those involved in other situations of surgical resection of the stomach: (i) subtotal/partial gastrectomy in bariatric surgery (gastric bypass, sleeve gastrectomy) and (ii) subtotal or total gastrectomy in surgical treatment of gastric cancer. The reduction of binge eating behaviors induced by gastrectomy could be due to a loss of stomach volume, but it is still unknown whether a gastric reservoir can affect food intake (19). Despite limited and low-quality evidence, Opozda et al. reported short to medium-term reductions in binge eating disorders and related behaviors after gastric bypass (20). Moreover, the same beneficial effects on eating behavior and hedonic component of taste perception have been also reported both after gastric bypass and sleeve gastrectomy in obese patients (21). Inversely, Mean and al. reported eating disorders after bariatric surgery in a systematic review (22). These post-bariatric eating disorders often existed before the surgery (binge eating disorder, loss of eating control, compulsive eating behavior), and are associated with less weight loss and/or more weight regain. Although binge eating may be anatomically impossible due to gastric volume restriction after surgery, other type of compulsive eating disorders may emerge, like grazing, rumination, etc. It is currently unclear whether any bariatric procedure leads to long-term improvement of eating behavior in some patients whereas (re)emerging of these in others.

As a consequence of gastric surgery, changes in gut-derived factors may mediate eating behavior changes. Serum ghrelin levels, orexigenic hormone, decreased to 10% to 20% of the preoperative level immediately after total gastrectomy in gastric cancer patients (23). Moreover, Taguchi et al. reported that fasting and postprandial elevation of glucagon-like peptide-1 (GLP-1) levels, anorexigenic hormone, were partly responsible of decreased food intake in the early postoperative period in rats after total gastrectomy (24). In their pilot clinical study, authors also reported that plasma fasting GLP-1 levels in patients with gastric cancer were increased greatly after total gastrectomy (on postoperative day 1). Furthermore, GLP-1, ghrelin, Peptide YY, neurotensin and oleoylethanolamide also mediate changes in eating behavior following gastric bypass and sleeve gastrectomy (25–27). Eating behavior changes after bariatric surgery, through increased taste and olfactory sensitivity, meal-size aversions and reduced hunger, have been recently reported (28).

**Figure 3:**
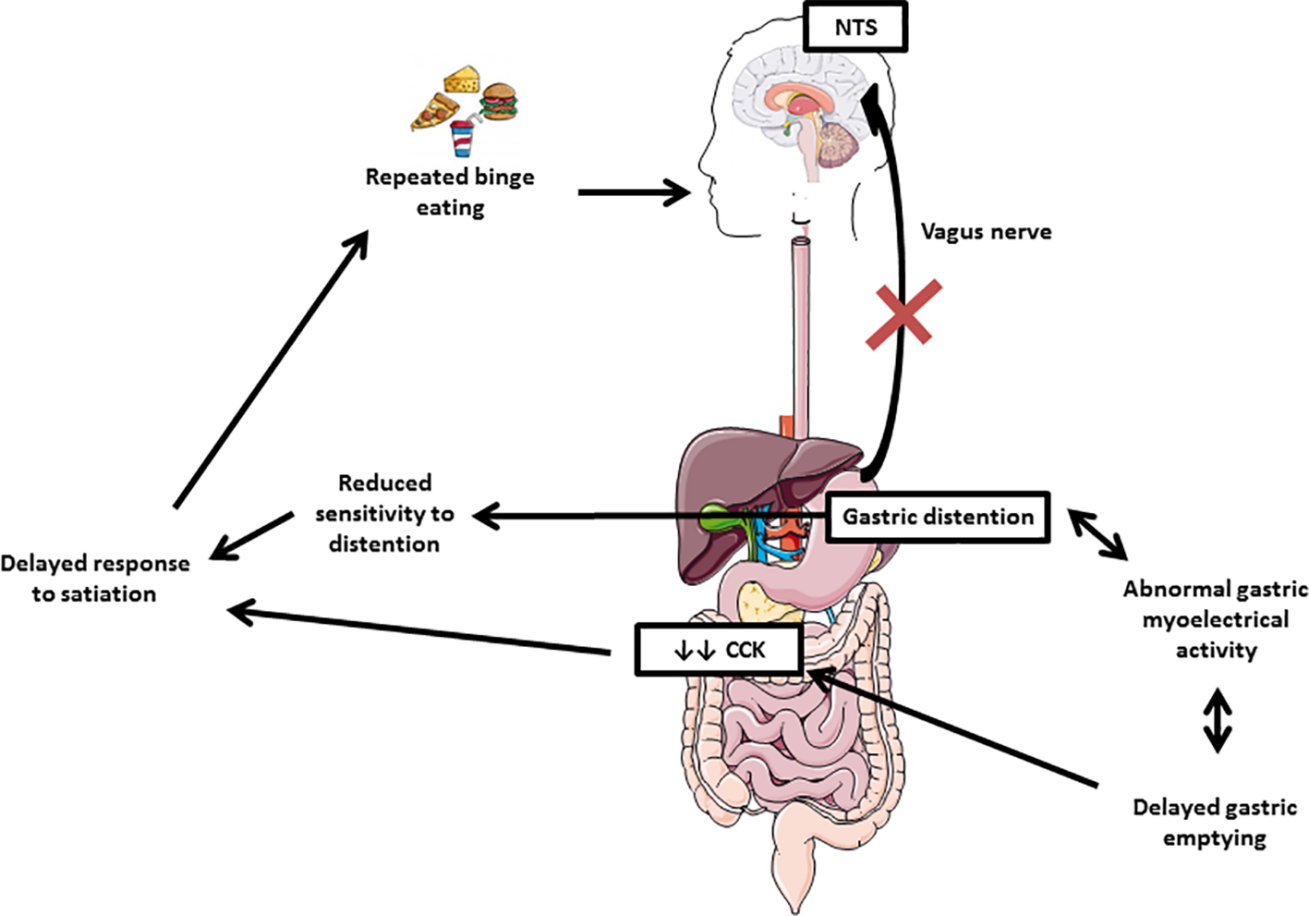
Gastrointestinal dysfunctions in bulimia nervosa. Abnormal gastric myoelectrical activity in patients with BN might result in (or coincide with) delayed gastric emptying leading to reduced postprandial cholecystokinin (CCK) release, which affects satiation. Impaired activation of the afferent vagus nerve that carries signals from the gut to brain areas (nucleus of the solitary tract, NTS) could also be involved in this pathophysiology.

Few old studies suggest a role for gastric motility alterations in the development and/or maintenance of BN. Recently, Van Dyck et al. reported a delayed response to satiation and abnormal gastric myoelectrical activity measured by electrogastrography before and after ingestion of noncaloric water, in 29 patients with BN and binge eating disorders vs controls (29). Moreover, patients with BN exhibit larger gastric capacities (30), reduced sensitivity to gastric distention (31), delayed gastric emptying (32), decreased post-prandial cholecystokinin (CCK) (32), and reduced gastric relaxation reflex (33). These gastrointestinal dysfunctions are summarized in **Figure 3**. Abnormal gastric myoelectrical activity in patients with BN might result in (or coincide with) delayed gastric emptying leading to reduced postprandial CCK release, which affects satiation. Impaired activation of the afferent vagus nerve that carries signals from the gut to brain areas (i.e., nucleus of the solitary tract, NTS) could also be involved in this pathophysiology (34). Interestingly, these gastrointestinal dysfunctions could also precede eating disorders symptoms, but unfortunately, this past information was not well documented for this patient. Further studies are needed to investigate the relationship between digestive hormones profil and the gastric myoelectrical activity during binge eating in BN.

Moreover, partial or total gastrectomy can affect gut microbiota composition, either in obese patients undergoing bariatric surgery (35) or in patients with gastric cancer after surgical treatment (36). Alterations in gut microbiota were significantly associated with reduced hedonic eating after sleeve gastrectomy in 8 obese women (37). This result suggests that surgically induced perturbations in gut microbiota-brain axis may play an important role in eating behavior. Further analyses in larger cohorts are needed. Finally, gastrectomy has an important psychological impact which may influence eating behavior. Interestingly, Hallowell et al. assessed the psychosocial impact of undergoing prophylactic total gastrectomy to manage the risk of Hereditary Diffuse Gastric Cancer (HDGC) in 27 adults though qualitative interviews (38). Three years postoperatively (median), all patients reported a loss of appetite and of hedonic eating.

To our knowledge, we reported for the first time the case of a patient who recovered from her eating disorder after a total gastrectomy following gastric necrosis. The present case report is limited by the lack of biological parameters, such as serum gut-derived hormones levels (ghrelin, GLP-1), faecal microbiota analyses, before and after gastrectomy. However, these biological samplings are not done routinely in our Nutrition Department. In the same way, we did not assess eating behavior using validated scales.

In conclusion, understanding the biological mechanisms mediating the eating behavior improvement engendered by total gastrectomy is challenging. Further studies are needed to assess postoperative changes in gut-brain axis including gut-derived factors such as gut hormones, microbiota, and neural signals acting peripherally and centrally upon homeostatic and hedonic brain regions.

## Data Availability Statement

The original contributions presented in the study are included in the article/supplementary material; further inquiries can be directed to the corresponding author.

## Ethics Statement

Written informed consent was obtained from the patient for the publication of this case report.

## Author Contributions

PD and NAi observed the patient and collected data. NAc collected additional information about the clinical case and wrote the paper. SG and MC critically reviewed the paper. All authors contributed to the article and approved the submitted version.

## Conflict of Interest

The authors declare that the research was conducted in the absence of any commercial or financial relationships that could be construed as a potential conflict of interest.
